# Recommendations on Postoperative Activities After Abdominal Operations and Incisional Hernia Repair—A National and International Survey

**DOI:** 10.3389/fsurg.2021.713138

**Published:** 2021-09-29

**Authors:** Sebastian Schaaf, Robert Schwab, Christoph Güsgen, Tim O. Vilz, Arnulf Willms

**Affiliations:** ^1^Department of General, Visceral and Thoracic Surgery, German Armed Forces Central Hospital Koblenz, Koblenz, Germany; ^2^Department of General, Visceral, Thoracic and Vasular Surgery, University Hospital Bonn, Bonn, Germany

**Keywords:** abdominal surgery, hernia surgery, postoperative activity, physical strain, incisional hernia, enhanced recovery (ER)

## Abstract

**Background:** There is no conclusive data on postoperative recommendations after abdominal and hernia surgery, and there is significant variation in the literature on that question. Thus, this study evaluates the status quo of recommendations of postoperative activity restriction after abdominal surgery.

**Materials and Methods:** A national (German) and international survey of general surgeons on postoperative recommendations after abdominal and hernia surgery was pooled and analyzed.

**Results:** A total of 74.6% recommended postoperative reduced activity for 2 weeks or less after laparoscopy. For midline laparotomy, 48.8% considered a reduced activity of 4 weeks or less to be sufficient. A majority from the national survey recommended more than 4 weeks instead (60.2%), whereas only 31.5% from the international survey did so (*p* = 0.000). In the pooled analysis, 258 of 450 (57.3%) rated 4 weeks or less suitable. However, the recommendations differed significantly between the surveys (4 weeks or less: a national survey, 47.1% vs. international survey, 64.6%; *p* = 0.000).

**Conclusion:** There was substantial variation in the given recommendations. However, we found no evidence against immediate mobilization, reduced physical activity, and lifting for up to 2 weeks after laparoscopic surgery and for up to 4 weeks after open abdominal surgery and open incisional/ventral hernia repair in uncomplicated and standard cases. There might be individual and socioeconomic benefits to allow patients to return to their whole personal level of activity and work without putting them at risk of complications. Due to lack of evidence, both retrospective and prospective, controlled studies are in need to develop reliable recommendations.

## Introduction

The most frequent questions of patients after abdominal and hernia surgery are related to posthospital behavior, postoperative activity, weight-bearing, and return to work. These questions have become even more critical since the overall length of hospital stay has reduced, and postoperative recovery algorithms are standard nowadays (1).

Nevertheless, there is uncertainty to answer those questions, as the recommendations in the scientific literature are very heterogeneous concerning the posthospital course (2). Thus, it is likely that the advice given to patients is somewhat arbitrary. Probably, the recommendations on postoperative activity might be too restrictive (2).

That concern has risen as there is a good body of evidence on the postoperative activity following inguinal hernia repair (3). It has been shown that restrictive advice after open and endoscopic inguinal hernia repair is not justified (4, 5). The available data clearly does not show higher complication or recurrence rates, even when activity has not been restricted at all or only for a couple of days after surgery (4, 5). Thus, current guidelines on inguinal hernia surgery state that patients should be encouraged to return to their average level of activity as soon as possible according to their level of pain (3).

Despite the conclusions drawn from inguinal hernia surgery data, might not be transferred to the specific situation of abdominal or incisional hernia surgery without caution, it still raises the question of whether the recommendations given are overcautious. A significant concern is that early or too progressive postoperative strain on the abdominal fascia might impair fascial healing and lead to higher incisional hernia rates or even burst abdomen. That is neither substantiated by biomechanical research nor observational study data (2).

Excessive precautionary measures are not only conducive to patient recovery but can also lead to significant socioeconomic damage.

As there is no conclusive data on postoperative recommendations after abdominal and hernia surgery and there is significant variation in the literature on that question, the data from previously published national (2) and international surveys (6) have been put together to evaluate further the status quo of recommendations of postoperative activity restriction after abdominal surgery.

## Materials and Methods

We included every German hospital listed with a surgical department on June 30, 2016, in a nationwide registry for the national survey. The department heads were asked to complete a questionnaire on their practice of postoperative recommendations on physical activity and strain after abdominal surgery. A brief description of that survey has already been published in the review by Güsgen et al. (2).

Following the national survey, we conducted an international study at the 41st Annual International Congress of the European Hernia Society (EHS) in Hamburg (September 11–14, 2019). The attendees were asked to complete a different questionnaire focused on rating proposals of postoperative reduction (abdominal and hernia surgery) of physical strain. The study was described in detail previously (6).

The national and international survey data were pooled and descriptively analyzed with Excel (vs. 2016, Microsoft, Redmont, Washington, USA) and SPSS (vs. 20, IBM, Armonk, New York, USA). Differences between the ratings were tested for significance with the chi-squared test. The level of significance was set to *p* = 0.05.

## Results

### National Hospital Survey on Given Recommendations of Postoperative Activity or Strain

A total of 1,078 German hospitals were asked to complete the questionnaire. A total of 386 of them (35.5%) responded and answered the questions. Some of the following results were mentioned briefly in the review article by Güsgen et al. (2).

**Figure 1:**
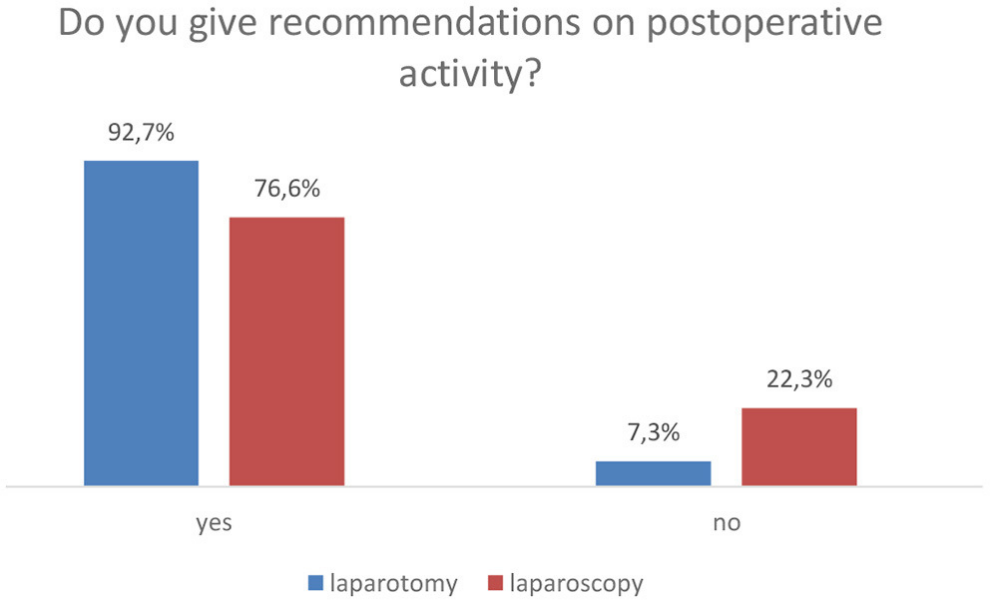
Comparison of whether recommendations of postoperative activities are given to patients in case of laparotomy and laparoscopy. n/a, not answered. *N* = 385, *p* = 0.000 (chi-squared test).

Most of the surgeons give recommendations on postoperative activity (Figure 1). Approximately 63% gave those recommendations written in the medical report on discharge and discussed them with the patients, and 31% discussed it with the patients only, and 1% gave it in the written report. Only 9.6% gave their recommendations based on the scientific data, 27.3% based on expert opinions, and 7.8% based on their own experience. The remaining (55.3%) did not mention the reasons for their recommendations.

**Figure 2:**
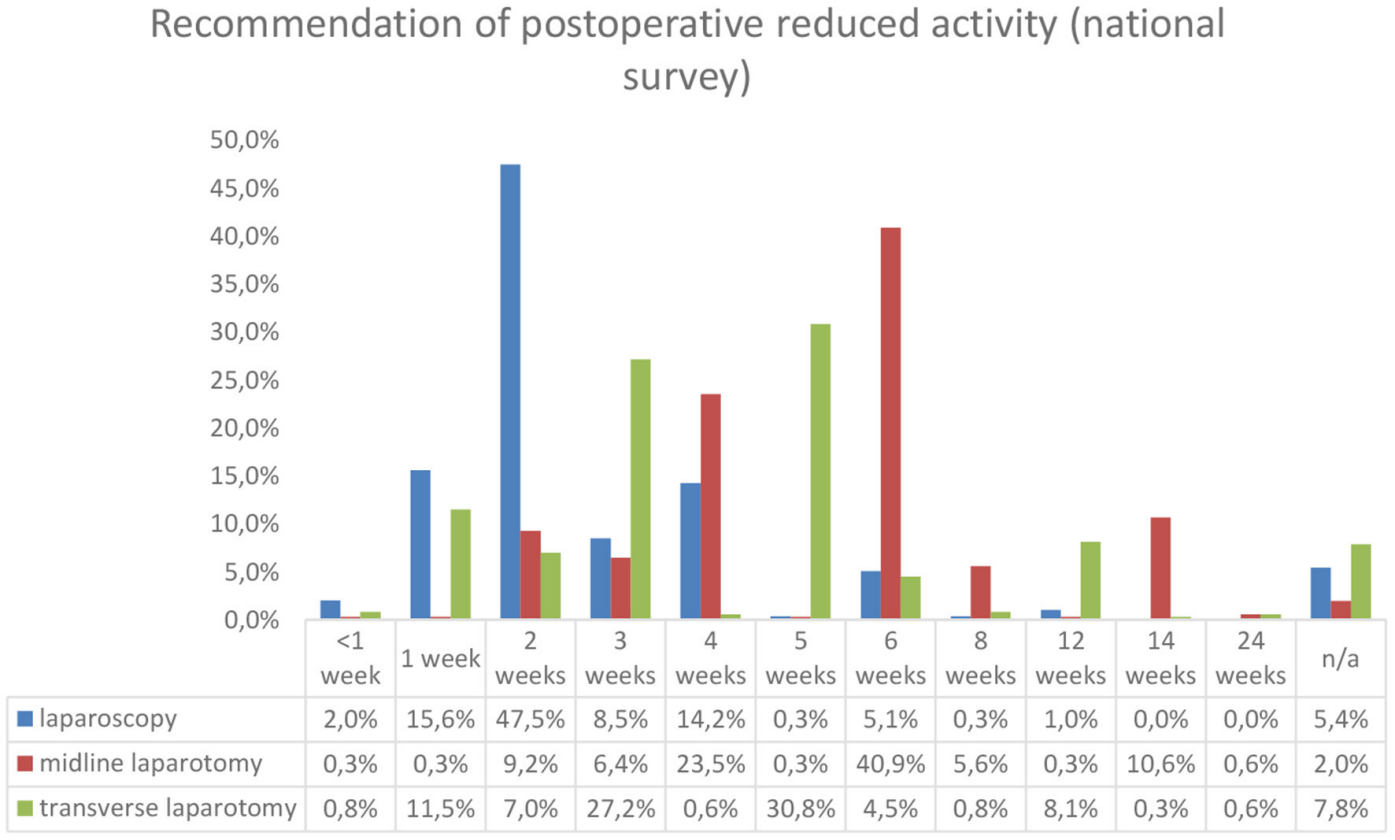
Duration of postoperative recommendations. *N* = 295 for laparoscopy and n = 357 for midline and transverse laparotomy.

The duration of recommended postoperative reduced activity is given in Figure 2.

**Figure 3:**
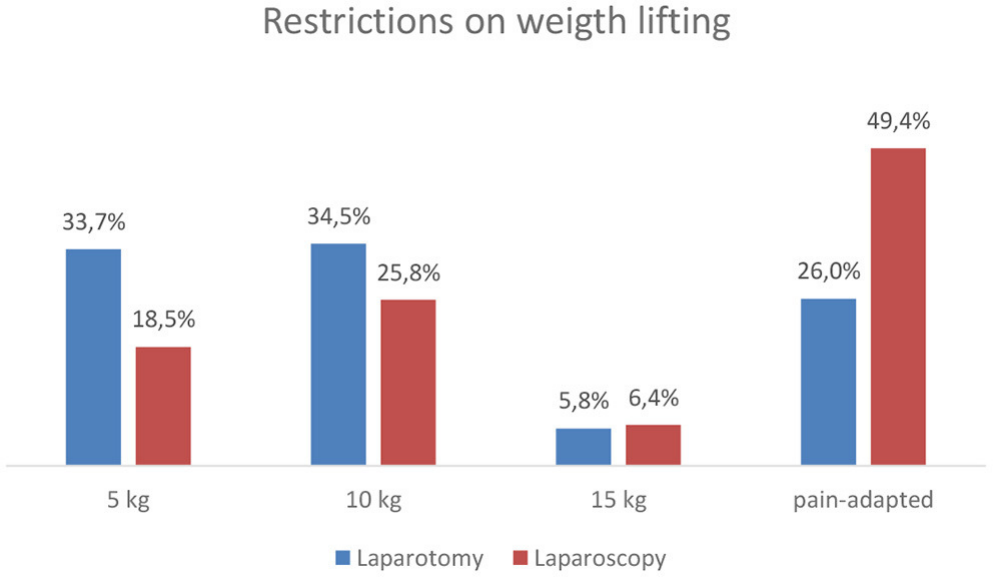
Given recommendations on weight lifting after laparotomy and laparoscopy.

The majority of the hospitals that completed the questionnaire reported giving recommendations on weight lifting after abdominal surgery (93.8% for laparotomy and 81.3% for laparoscopy). Figure 3 provides an overview of the results. After laparoscopic abdominal surgery, 49.5% recommends weight lifting adapted to individual pain sensation, whereas 26.0% did so for laparotomy. About one-third recommended 5 or 10 kg after laparotomy.

**Figure 4:**
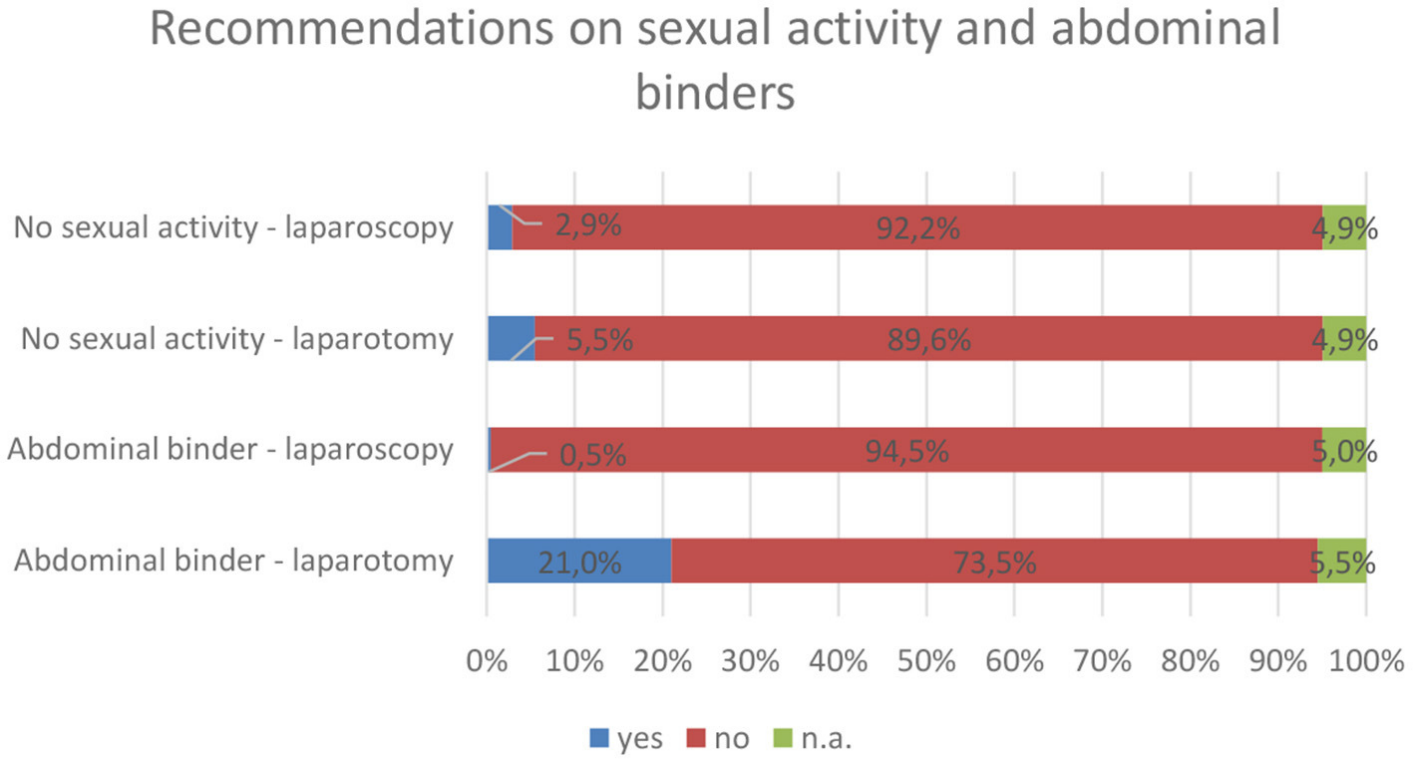
Overview of recommendations on abdominal binders and sexual activity. Of those who recommend abdominal binders after laparotomy, 20.0% advise patients to wear them for 2 weeks, 23.5% for 4 weeks, and 27.2% for 6 weeks. n.a., not answered.

An overview of the survey results for the use of abdominal binders and recommendations on sexual activity is shown in Figure 4. Abdominal binders were reported to be recommended in 21.0% after laparotomy for 2–6 weeks and only in 0.5% after laparoscopic procedures. Regarding sexual activity, in 90.0/92.2% (laparotomy/laparoscopy), no specific recommendation was given. However, if such a recommendation was given, 2 weeks of celibacy were considered appropriate.

For sports, 80.0% of the participating hospitals reported giving recommendations to laparotomy patients (6 weeks 23.9%, 4 weeks 23.4%, and 2 weeks 15.1%, the remaining did not further specify). For laparoscopy, fewer hospitals (56.4%) recommended refraining from sports (mostly 2 weeks).

Only one-third of the hospitals stated sick leave, irrespective of laparotomy or laparoscopy. In most cases, the duration of recommended sick leave was not specified. Mostly 2 weeks or less were recommended after laparoscopy. The recommended durations after laparotomy were very heterogenous but mostly substantially longer than 2 weeks.

### International Hernia Surgery Expert Survey on Postoperative Activity or Strain

The survey results were published previously in detail elsewhere (6). A total of 127 participants took part in the survey and rated given proposals for postoperative rest or refrain from physical activity.

Those proposals were 2 weeks for laparoscopy and 4 weeks for laparotomy (transverse and midline) or incisional hernia surgery (mesh-augmented reconstruction with sublay, intraperitoneal onlay mesh (IPOM), onlay, or complex techniques like component separation plus sublay).

#### Abdominal Surgery

For laparoscopy, 2 weeks were rated appropriate (57.5%) or even too long (36.2%). Put together, 93.7% agreed on a resting interval of up to 2 weeks.

Regarding open abdominal surgery, the ratings of 4 weeks of resting after midline or transverse laparotomy were similar: 56.7/52.8% (midline/transverse) considered that appropriate or too long 11.8% (midline and transverse). That led still to a majority of 68.5/64.7% (midline/transverse) that regards 4 weeks of rest after laparotomy to be sufficient. However, it should be noted that a third (31.5% midline and 29.9% transverse) rated 4 weeks as too short and recommended a substantially longer period of 7.0 ± 1.6/6.6 ± 1.1 (midline/transverse) weeks, instead.

#### Hernia Surgery

The results for sublay or IPOM repair were similar. The given period of 4 weeks was rated appropriate in 55.1/52.0% (sublay/IPOM) and too long in 13.4/21.3% (sublay/IPOM). Summed up, 68.5/73.3% (sublay/IPOM) of the survey participants agreed on up to 4 weeks. However, like for laparotomy, a substantial proportion of 31.5/22.8% (sublay/IPOM) considered that too short and proposed 7.0 ± 1.7/7.4 ± 1.9 (sublay/IPOM) weeks.

The results for onlay repair were somewhat different, as only 44.9% rated 4 weeks appropriate or too long, whereas 37.0% considered it too short and proposed 7.4 ± 1.9 weeks. Moreover, 18.1% did not rate that technique.

For complex hernia repair techniques, most participants were convinced that 4 weeks were too short (47.2%); thus, a period of 7.2 ± 2.3 weeks was recommended. A total of 37.0/7.1% rated that those 4 weeks were appropriate or too long.

### Pooled Analysis of National and International Survey Results

**Figure 5:**
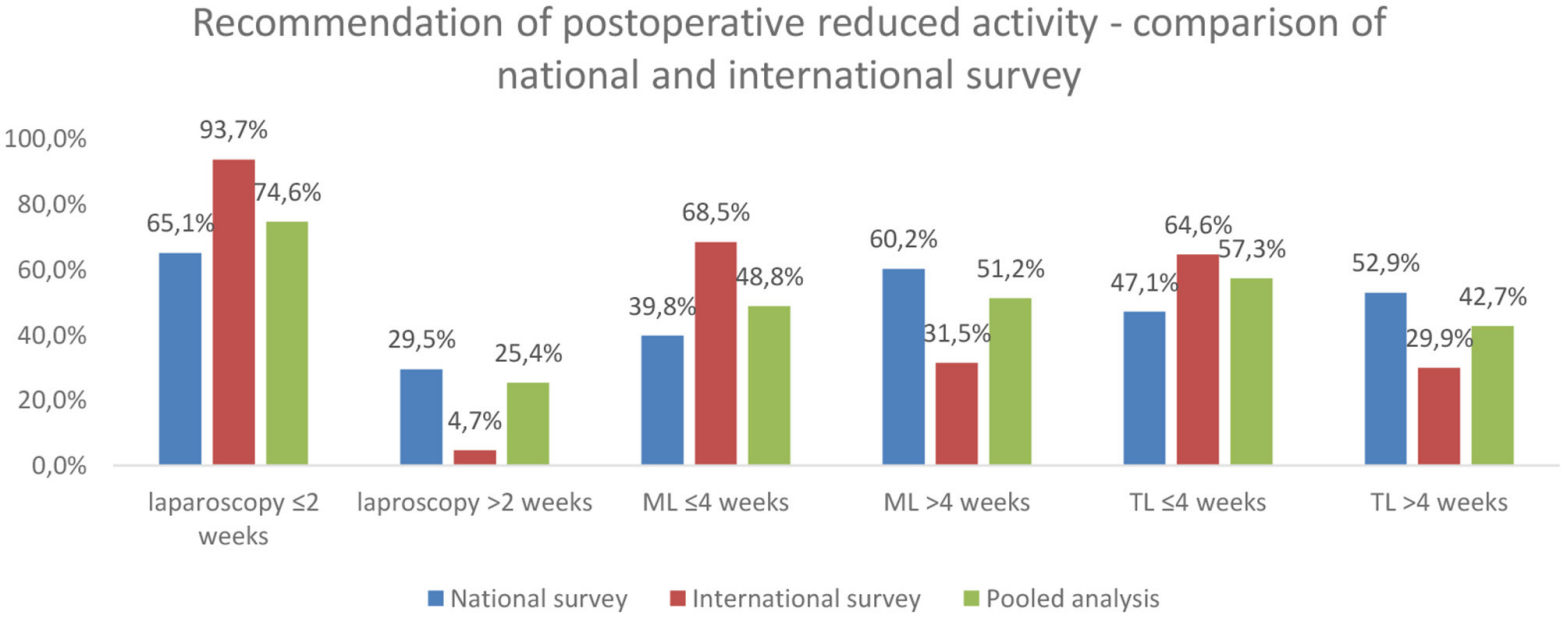
Comparison of survey results. The differences were statistically significant (*p* = 0.000; chi-squared test).

The pooled analysis results of both surveys are shown in Figure 5. In total, 299 of 401 (74.6%) recommended postoperative reduced activity for 2 weeks or less after laparoscopy. A more extended period was recommended in the national survey in 29.5 vs. only 4.7% in the international survey (*p* = 0.000).

For midline laparotomy, 230 of 471 (48.8%) considered a reduced activity of 4 weeks or less to be sufficient. However, a majority from the national survey recommended more than 4 weeks instead (60.2%), whereas only 31.5% from the international survey did so (*p* = 0.000).

Considering the recommendations on transverse laparotomy, the results were similar. In the pooled analysis, 258 of 450 (57.3%) rated 4 weeks or less suitable. However, the recommendations differed significantly between the surveys (4 weeks or less: a national survey, 47.1% vs. international survey, 64.6%; *p* = 0.000).

## Discussion

Reliable data concerning evidence-based recommendations for patients considering postoperative strain after abdominal or hernia surgery are scarce. A national survey of surgical departments revealed that 94% give such advice to patients (2). However, the data also showed that fewer than 10% justify their recommendations based on the available scientific data, and more than 50% did not report based on their recommendations.

When the data from the national and the international surveys were compared, we found that 75% agree on a postoperative reduced activity after laparoscopy of 2 weeks or less (2, 6). Regarding midline or transverse laparotomies, the results were inconclusive, as about one-half decided on 4 weeks or less of postoperative reduced activity and the other half on more than 4 weeks. It is worth mentioning that we also found substantial variance between the answers given in the national and in the international survey, which leads to the conclusion that the participants of the latter, experts in hernia surgery, attendees of the 41st Annual Meeting of the European Hernia Society, agreed to a more significant proportion on shorter intervals of postoperative reduced activity. Similar results were shown in a survey of 44 surgical departments by Paasch et al. (7).

Why is there so much variation in the recommendations on postoperative reduced activity after abdominal and hernia surgery? The risk of recurrence or development of incisional hernias, the fear of causing mesh-related complications, or just prolonged pain is typically mentioned as theoretical considerations and can result in arbitrary recommendations. Those risks cannot be substantiated by published data (8, 9). For inguinal hernia repair, it has been shown that early and progressive strain or the immediate return to physical work is not associated with hernia recurrence. Consequently, the recommendations for the postoperative recovery to total physical activity have changed in inguinal hernia surgery and are pretty progressive nowadays (3).

### Intraabdominal Pressure, Wall Tension, and Physical Activity

From a biomechanical perspective, the intraabdominal pressure rise associated with physical activity or lifting seems to be a critical factor as it directly increases the wall tension following Laplace's law. It has been shown that the increase of intraabdominal pressure depends on the amount of weight and how it is lifted (10). However, it has also been shown that even slowly lifting weights of up to 50 kg led only to a negligible intraabdominal pressure rise (11).

On the contrary, a substantial rise in intraabdominal pressure was reported for actions like coughing, defecation, or vomiting (11). Unlike controlled postoperative mobilization and return to normal daily activities, those physiological and involuntary cannot be prevented. The national survey results also showed more than 90% of the participating surgical departments advise their patients on postoperative sexual activity (2). That seems problematic since that aspect dramatically impacts the quality of life, and Valsalva's maneuver was found to double intraabdominal pressure (12).

Put those arguments together, leading to fascial shear stress at the sutured incision for the principle biomechanical factor. It has been shown that the sutured fascia has substantially reduced resistance compared to healthy fascia no matter what suture material was used (13, 14). However, fascial tissue heals quickly due to increased fibroblast activity, collagen synthesis, and contraction (15, 16). After 28–30 days, there was no difference in biomechanical resistance detectable between native and incised fascia, though healing was uncomplicated (e.g., wound infection) (14, 15). For mesh-augmented fascial incisions (mesh-augmented inguinal hernia repair), it has even been shown that fascial resistance is comparable to native fascia immediately (4).

### Factors of Incisional Hernia Development

The biomechanical strain on the incised fascia cannot be held only responsive for incisional hernia development. The main argument against this is that most incisional hernias or recurrent incisional hernias do not develop immediately after surgery; merely 50–60% occur after 1 year or more postoperatively, and only less than that 10% develop within the first year (2, 14, 17, 18).

Thus, other factors unaffected by the postoperative activity or straining might also be involved. An important factor is the impaired collagen metabolism (19, 20). In hernia specimens, a higher amount of immature and biophysically inferior collagen III and an altered composition and activity of matrix metalloproteinases were detected (21, 22). Such alterations were also found in connective tissue unrelated to hernias; hence, an underlying genetical disorder, also unaffected by postoperative physical activity, is likely (20).

Also, numerous patient-specific factors include higher age (>45 years), obesity, and a thicker subcutaneous fatty tissue layer, multiple abdominal surgeries, malign disease and chemotherapy, aortic aneurysms, diabetes, smoking, or positive history of hernias elsewhere, are associated with incisional hernia development (9, 14, 18, 23–25).

Moreover, also surgical-technical aspects are of relevance for the risk of incisional hernia development. Such is the technique of abdominal wall closure (fascial closure with small-bites technique, long-term absorbable running suture, suture-to-wound-length-ratio > 4:1) (9), and surgical site infections (26). The state of wound healing within the first 30 days after surgery might have a substantial effect. According to Franz and Pollock (27, 28), early scar breakdown due to impaired wound healing is the key factor in the development process of incisional hernia and burst abdomen. Hence, any measure to prevent any factors that might hamper proper wound healing needs to be taken. Thus, prophylactic implantation of meshes after laparotomies is under investigation, as promising results in terms of reduced incisional hernia rates have been shown in high-risk patients (29).

### Implications for the Clinical Routine

One of the significant treatment goals in abdominal and hernia surgery is restoring the abdominal wall integrity and its load-bearing capacity. Biomechanical considerations can help to achieve these goals in the long term (30, 31). It could be shown that there is a considerable variance and substantial uncertainty in terms of postoperative recommendations on physical activity and strain (2, 6, 7, 9, 32). In addition, our national and international surveys found substantial differences between the recommended duration of postoperative reduced activity for laparotomies and laparoscopy.

Abdominal binders were recommended by only 21% and only after laparotomies in the national survey. The effect of abdominal binders after abdominal surgery is unclear, though the acceptance by the patients is subjectively high (7, 33, 34). There might be a beneficial effect in reducing pain and facilitating mobilization after open abdominal or hernia surgery (35–37). For laparoscopic procedures, the data shows conflicting results (38, 39).

Dietz et al. (40) recommended a review on incisional hernia techniques postoperative reduced activity or weight-lifting restrictions for 3–6 weeks, though they also stated there is not enough evidence to substantiate any binding recommendation.

We are convinced to summarize there is insufficient data to justify a recommendation of reduced strain or activity after uncomplicated abdominal and incisional hernia surgery longer than 4 weeks to reduce the risk of incisional development/recurrence. Furthermore, if the available study data and the findings of our surveys are compared, we suppose the postoperative duration of reduced activity and strain is overestimated by a substantial proportion of the survey participants, particularly in the German collective.

However, that directly affects the duration of sick leave or return to work and indirect healthcare costs. Return to work or regular physical activity is one of the most critical outcome factors after the survival of the patients (41). The general practitioners are highly likely to follow the advice given by the operating surgeons (42).

Comprehensive guidelines on postoperative behavior, especially refraining from activity or lifting, are available neither for abdominal surgery, in general, nor for specific procedures (e.g., appendectomy, cholecystectomy, and colorectal) (2). An expert panel developed recommendations on convalescence in the Netherlands; however, those recommendations have to be evaluated, and there is no data yet available (43). Those recommendations were for inguinal hernia repair and laparoscopic procedures (appendectomy and cholecystectomy) 2 weeks until the resumption of total activity or return to work, which is in line with our findings. For open abdominal surgery (colonic resection), 4–8 weeks were somewhat longer than proposed by participants of our international survey (6) and similar to the ratings in our national survey (2).

In Germany, the German Society for General and Visceral Surgery (surgical working group hernia, CAH) stated postoperative reduced activity after hernia surgery. The recommended duration of decreased activity or refrain from heavy physical labor does not exceed 4 weeks for all surgical procedures, but complex hernia repair including component separations (4–6 weeks). It is remarkable, though, restriction of normal daily activities is not recommended at all. Those recommendations are in line with our findings and are depicted in Table 1.

**Table 1:** Recommendations of postoperative activity after abdominal and hernia surgery were modified according to Güsgen et al. (2).

**Recommendation**	**Laparoscopic procedures**	**Combined or hybrid approaches**	**Open abdominal/hernia surgery**
**Resting**	None	None	None
**Straining and physical activity**				
Activities of daily life	Immediately	Immediately	Immediately
Rehab and guided physical therapy	Immediately after wound healing	Immediately after wound healing	Immediately after wound healing
Sports and sexual activity	After wound healing, pain is the limit	After 2–4 weeks, pain is the limit	After 3–4 weeks, pain is the limit
**Sick leave**				
Office job/light physical labor	1–2 weeks	2–4 weeks	4 weeks
Heavy physical labor	2–3 weeks, depending on pain	3–4 weeks, depending on pain	4 weeks, depending on pain
**Abdominal binder**	Offer during hospital stay, afterward depending on pain	Offer during hospital stay, afterward depending on pain	Offer during hospital stay, afterward depending on pain

*In case of a complicated postoperative course, impaired wound healing or extensive/complex procedures longer periods might be necessary based on a decision of an individual surgeon. Therefore, wound healing with this is considered as the day of suture removal*.

## Limitations

It is essential to mention that implications and conclusions drawn from the results of this study need to be interpreted with caution. Survey results are subjective opinions and likely to be biased in some ways. Also, there was no structured process established to reach a consensus among the participants. Thus, the results merely provide an overview of the spectrum of recommendations given by active surgeons and reflect the available literature, which can only be qualified as the lowest level evidence.

## Conclusion

Put together, there is definitively a lack of evidence regarding postoperative recommendations on return to total physical activity or work and lifting after abdominal and hernia surgery. Moreover, that is represented by the substantial variance in evaluating that topic by both international and national (German) surgeons. When the available literature on that topic, fascial healing, and incisional hernia development are taken into account, the given recommendations likely cause too long physical activity restrictions. Thus, we see no justification for not to mobilize immediately after surgery, reduce physical activity and lifting for up to 2 weeks after laparoscopic surgery and inguinal hernia repair, for up to 4 weeks after open abdominal surgery and open incisional/ventral hernia repair. That applies only to uncomplicated postoperative courses, and different decisions might still be reasonable in exceptional cases.

It seems there is a great potential to allow patients to return to their full individual level of activity and work without putting them at risk of short- or long-term complications. Also, that could have a beneficial impact in a socioeconomic context, as sick leave durations and healthcare costs might be reduced.

Therefore, and due to the apparent lack of evidence in that field, retrospective evaluation of the given advice is necessary. Then, straightforward recommendations on postoperative convalescence can be developed and will need to be prospectively evaluated in a case-control setting.

## Data Availability Statement

The raw data supporting the conclusions of this article will be made available by the authors, without undue reservation.

## Author Contributions

SS performed the analysis and interpretation of the data. SS and AW created the draft of the manuscript. RS, CG, TV, and AW revised it critically for important intellectual content and were equally responsible for conceptual development of the work. All authors declare to have provided substantial contributions to the conception and design of the work.

## Conflict of Interest

The authors declare that the research was conducted in the absence of any commercial or financial relationships that could be construed as a potential conflict of interest.

## Publisher's Note

All claims expressed in this article are solely those of the authors and do not necessarily represent those of their affiliated organizations, or those of the publisher, the editors and the reviewers. Any product that may be evaluated in this article, or claim that may be made by its manufacturer, is not guaranteed or endorsed by the publisher.
